# Phonolite Material as Catalyst Support for the Hydrotreatment of Gas Oil and Vegetable Oil Type Feedstocks

**DOI:** 10.3390/ma15010386

**Published:** 2022-01-05

**Authors:** Héctor de Paz Carmona, Jakub Frątczak, Zdeněk Tišler, José Miguel Hidalgo Herrador

**Affiliations:** 1 ORLEN UniCRE a.s., Revoluční 1521/84, 400 01 Ústí nad Labem, Czech Republic; jakub.fratczak@orlenunicre.cz (J.F.); zdenek.tisler@orlenunicre.cz (Z.T.); jose.hidalgo@orlenunicre.cz (J.M.H.H.); 2 Department of Chemistry, Faculty of Agrobiology, Food and Natural Resources, Czech University of Life Sciences Prague, Kamýcká 129, 165 00 Prague, Czech Republic

**Keywords:** hydrotreating, co-processing, phonolite, natural materials, hydrotreated vegetable oil

## Abstract

Phonolite material has shown to be promising catalyst support for the deoxygenation of triglycerides. In this work, we continue with our previous research by synthesising and testing three acid-treated phonolite-supported Co-Mo, Ni-Mo and Ni-W catalysts for the hydrotreating of atmospheric gas oil and co-processing with rapeseed oil at industrial operating conditions (350–370 °C, WHSV 1–2 h^−1^, 5.5 MPa) in the continuous regime for more than 270 h. The phonolite-supported catalysts showed hydrotreating activity comparable with commercial catalysts, together with a complete conversion of triglycerides into n-alkanes. During co-processing, the Ni-promoted catalyst showed strong stability, with similar activity previous to the rapeseed oil addition. Our results enable us to evaluate the suitability of phonolite as catalyst support for the development of plausible alternatives to conventional hydrotreating catalysts for the co-processing of middle distillates with vegetable oils.

## 1. Introduction

In December 2019, the European Commission (EC) presented the Green Deal, a challenging commitment to convert the EU into the first climate-neutral continent. The Green Deal focused on several key principles related to the energy issues: Ensure a secure and affordable energy supply for the EU and prioritise energy efficiency, developing a power sector based on renewable sources. In this context, the production of biofuels and suitable catalysts play a significant role in achieving the highly demanding targets related to the production of renewable energy.

In the frame of biofuels, hydrotreated vegetable oil (HVO) is considered, together with FAME (fatty acid methyl esters), as one of the most promising biofuels [1,2,3,4]. HVO production by co-processing is considered the most attractive option, considering the current refinery infrastructure available [5]. This option produces low-carbon hybrid fuels and allows the gradual decarbonisation of fuels [6,7]. The co-processing has already been pointed to by numerous studies as plausible [8,9], even in the case of waste oils (waste cooking oils or animal fats) [10,11], which is in line with the circular economy action plan (CEAP) of the EU, “Less waste, more value”.

**Figure 1:**
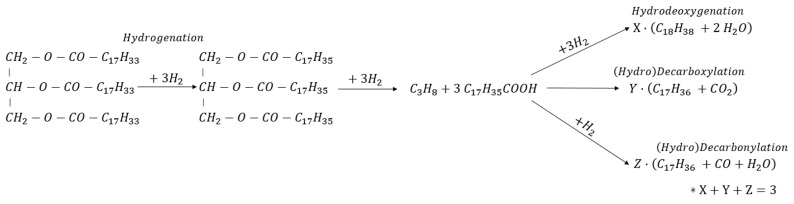
Triglyceride deoxygenation pathways. Tri-olein as a model molecule [12,13]. * The sum of the possible hydrotreating pathways is always three (i.e., X + Y + Z = 3).

The co-processing pathway combines hydrotreating of the middle distillate (including hydrodesulfurisation (HDS), hydrodenitrogenation (HDN) and hydrocracking), together with the deoxygenation (DO) of triglycerides into n-alkanes [12,13]. The triglyceride molecules are first hydrogenated during the hydrotreating, then broken to produce a propane molecule and three carboxylic acids. Depending on the operating conditions or catalysts used [14], the reaction continues according to three possible reactions: Hydrodeoxygenation (*HDO*) and (hydro) decarboxylation/decarbonylation (*DCO*). As shown in Figure 1, the main differences between these pathways are focused on the length of the n-alkanes (usually 17 and 18 carbon atoms for *HDO* and *DCO* pathways, respectively) and the side products. The *HDO* pathway lead to H_2_O production, accompanied by higher H_2_ consumption. On the other hand, *DCO* pathways lead to higher CO/CO_2_ production but lower H_2_ consumption.

In the frame of catalysts, the current commercial hydrotreating catalysts (i.e., NiMoSx or CoMoSx supported by Al_2_O_3_/SiO_2_ supports) are usable for co-processing. However, this is only when the triglyceride feedstocks are in the low range (0–10 wt.%) [15]. Co-processing options seem unaffordable for conventional catalysts at higher ratios, significantly increasing the deactivation ratio due to sulfur leaching and coking [16]. This collateral effect points to the need for catalyst development, looking for suitable catalysts that allow for increasing the biomass component. In this way, the current research focuses on developing sulfur-free catalysts [17] or new materials as catalyst supports that show higher stability during co-processing.

In this context, during the last five years, several studies have pointed to phonolite material (Ph) as an excellent and exciting material for industrial applications. To the best of our knowledge, most of these references are focused on several research areas, such as geology, geochemistry, or mineralogy [18,19,20]. Nevertheless, our previous studies indicated that this igneous rock is also suitable as a catalyst support or metal-adsorbent (Ca, Mg, K, P and Na), increasing its specific surface (from 5 to 167 m^2^/g) after an acid treatment [21]. In this way, the following studies were focused on the characterisation, and use of Ni-W Ph for the deoxygenation of animal fats and vegetable oils, obtaining a high rate of oxygen compounds decrease [22,23]. In line with these promising results, the subsequent studies focused on synthesising Co-Mo, Ni-Mo and Ni-W Ph catalysts for biofuels and platform chemicals production. In this sense, the co-hydrocracking of heavy Fischer-Tropsch waxes has been tested with vacuum gasoil [24] and the mesityl oxide reduction [25]. The results achieved show the suitability of phonolite as catalyst support. Thus, it is mandatory its further study it for biofuels production to obtain sustainable decarbonisation of fuels as EU directives demand.

This work is the following step in our research on the uses of phonolite material as suitable catalyst support. For this purpose, we synthesised and tested three acid-treated phonolites (Ph-HCl)-supported Co-Mo, Ni-Mo and Ni-W catalysts (Co-Mo/Ph-HCl, Ni-Mo/Ph-HCl, Ni-W/Ph-HCl) for the hydrotreating of atmospheric gas oil (AGO) and its blends with rapeseed oil (RSO: 5, 10 and 25 wt.%) at industrial operating conditions (350–370 °C, WHSV 1–2 h^−1^, 5.5 MPa). Our results enable us to evaluate the suitability of this natural material as catalyst support, resulting in a plausible alternative to conventionally hydrotreating catalysts.

## 2. Materials and Methods

### 2.1. Feedstocks

The middle distillate used for hydrotreatment and co-processing was commercial atmospheric gas oil (AGO), obtained from the atmospheric distillation of Russian export blend crude oil. The vegetable oil used for co-processing was food-quality commercial rapeseed oil (RSO) supplied by the Aro company. Both feedstocks together with their mixtures (AGO/RSO 95/5, 90/10 and 75/25 wt.%) were characterised using the following standards procedures: Density at 20 °C [26], the refractive index at 20 °C [27], sulfur (S—[28]) and nitrogen (N—[29]) content, acid number [30], elemental C, H analysis [29] and simulated distillation (Simdis—[31]). Standard refinery hydrotreating gas (97.5–99.5 vol% H_2_ plus 0.5–2.5 vol% CH_4_) was used as the H_2_ supply.

### 2.2. Catalysts Synthesis and Characterisation

Three acid-treated phonolites (Ph-HCl)-supported Co-Mo, Ni-Mo and Ni-W catalysts (Co-Mo/Ph-HCl, Ni-Mo/Ph-HCl, Ni-W/Ph-HCl) were synthesised using a co-impregnation method. The authors described the acid treatment used for phonolite modification in a previous article [21]. Nevertheless, the procedure is described briefly below.

The Keramost a.s. company supplied the phonolite raw material (Ph) in different particles sizes. First, the phonolite raw material was sieved using a Retsch AS300 to obtain a range of sizes of 0.224–0.560 mm fraction. Then, 20.0 g of Ph in that size fraction was dried at 120 °C for 12 h. Later, the Ph material was dealuminated using a 3.0 M HCl water solution in a batch reactor for 4 h at 80 °C (Ph/HCl ratio 1:10 wt.%/vol.%). The resulting product was filtered, washed with hot demineralised water and dried at 120 °C for 12 h again. Finally, Co-Mo/Ph-HCl, Ni-Mo/Ph-HCl and Ni-W/Ph-HCl catalysts (Ni/Co: 5 wt.%-Mo/W: 10 wt.%) were prepared by co-impregnation with an aqueous solution of metal precursors (nickel/cobalt nitrate hexahydrate, ammonium heptamolybdate and ammonium meta-tungstate), and the catalyst precursors were dried at 120 °C for 12 h and calcined in air at 450 °C during 6 h (1 °C /min).

After the synthesis was finished, fresh catalyst samples were characterised using several methods. X-ray fluorescence (XRF; S8 Tiger with an Rh cathode, Bruker AXS GmbH, Karlsruhe, Germany) and inductively coupled plasma spectroscopy (ICP-OES; Agilent 725; Agilent Technologies Inc., Santa Clara, CA, USA) were used to determine the bulk catalyst composition. X-ray diffraction (XRD; D8 Advance ECO, Bruker AXS GmbH, Karlsruhe, Germany; Cu K α radiation and λ = 1.5406 Å) was used to identify the composition of phases. The resolution of XRD was 0.02° and the step time was 0.50 s. The measurements were performed over a 2-theta range of 5° to 70° and evaluated by Difrac.EVA software (Bruker AXS GmbH, Karlsruhe, Germany) using the Powder Diffraction File Database (PDF 4 + 2018, International Centre for Diffraction Data, Newtown Square, PA, USA).

The textural properties were characterised by the mercury Porosimetry using AutoPore IV 9510 (Micromeritics Instrument Corporation, Norcross, GA, USA) and the nitrogen physisorption using an Autosorb iQ (Quantachrome Instruments, Boynton Beach, FL, USA). The specific surface area was calculated from the adsorption isotherm’s linear plot using the Brunauer, Emmett and Teller (BET) Method in the pressure range of 0.05–0.30 P/P_0_. The catalyst acidity and basicity were characterised by NH_3_-TPD and CO_2_-TPD, respectively, using an Autochem 2950 HP (Micromeritics Instrument Corporation, Norcross, GA, USA). For these analyses, the sample preparation consisted of heating to 500 °C (10 °C/min) in a He flow of 25 mL/min for 30 min, followed by cooling down to 100 °C in a He flow of 25 mL/min. After this procedure, the temperature increased to 500 °C (15 °C/min) to obtain TPD curves from 100 to 500 °C. A TCD detector monitored the changes in the gas concentration. Finally, the catalyst morphology was studied by a scanning electron microscope JSM-IT500HR from JEOL (JEOL, Tokyo, Japan). The voltage was 15 kV, and the detector used was a secondary electron (SED). Samples were converted with 5 nm of gold to make them conductive and prevent charging the samples in the microscope.

### 2.3. Experimental Setup and Catalytic Tests

**Figure 2:**
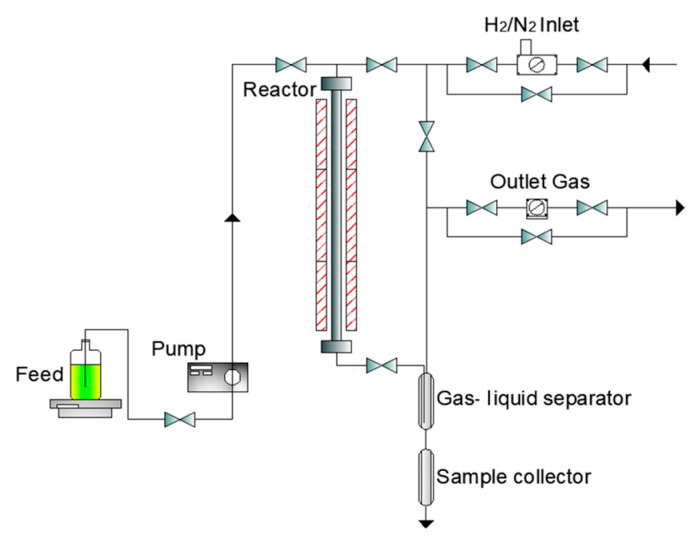
Schematic diagram of the used bench-scale unit for hydrotreating experiments (experimental facility of ORLEN UniCRE a.s.).

The hydrotreating experiments were carried out in a bench-scale unit with a stainless-steel reactor (internal diameter 17 mm). Three thermocouples were located in a 5 mm outer diameter thermowell for measuring and controlling the catalyst bed temperature. The heating system was composed of a triple-zone electric heater. During co-processing stages, the reactor outlines can be heated at 50 °C to avoid plugging problems from unconverted triglycerides-hydrotreated products. The unit was located in the experimental facility of ORLEN UniCRE a.s. (Litvínov-Záluží, at the Czech Republic). Figure 2 shows a schematic diagram of the unit used.

The catalyst experiments were executed similarly to the authors’ experiments for testing catalytic materials in a continuous regime [32]. The procedure is described as follows. Each hydrotreating experiment used 15.0 g of the Ph-HCl catalyst (Co-Mo/Ph-HCl, Ni-Mo/Ph-HCl or Ni-W/Ph-HCl). The catalyst bed was composed of three parts of 5.0 g of catalysts diluted with fine carborundum (SiC, 0.1 mm) in ratios of 1:1, 1:1 and 1:2 (vol:vol). The most diluted part (1:2) was situated at the top of the reactor, where most exothermic reactions were expected. The catalyst concentration increased along the catalyst bed, maintaining the reactor temperature profile and isothermal state in this loading procedure. After catalyst loading, the reactor was flushed with N_2_ (600 NL h^−1^, 100 kPa, and 25 °C) for 2 h and the pressure was increased to 9.0 MPa to perform the leak test. After the leak test, the pressure was reduced to 5.5 MPa, and the gas was changed to H_2_ (15 NL h^−1^). Then, the catalyst sulfidation started. For that purpose, the catalyst bed was heated from an ambient temperature to 150 °C at 50 °C h^−1^. After 2 h at those conditions, a mixture of AGO and dimethyl disulphide (DMDS: 3 wt.%) was fed to the catalyst bed, and the temperature increased to 350 °C (15 °C h^−1^). After 7 h at those conditions, the sulfidation procedure finished, the feedstock was changed to AGO (30.0 g; WHSV 2.0 h^−1^) and the H_2_ flow rate was increased to 30 NL h^−1^. Each catalyst was tested using different operating conditions (reaction temperatures, WHSV and feedstocks). Table 1 summarises the operating conditions of the catalyst experiments, described in chronological order during testing.

### 2.4. Product Characterisation

After the hydrotreating reaction, a gas/liquid separator divided the product into phases. The liquid product mainly consisted of hydrotreated gas oil and water during co-processing. This liquid product was sampled and weighed every 4 h for mass balancing. Density at 20 °C [26] and a refractive index [27] at 20 °C was determined for all samples taken (organic phase only) to monitor catalyst activity and unit processing. In this sense, the steady state of the unit was achieved when the density and refractive index were stable during processing. The hydrotreated gas oil collected at a steady state was analysed using similar techniques employed for the feedstocks and IR-attenuated total reflectance (ATR) during co-processing. This analytical method allowed the evaluation of RSO triglycerides’ conversion to n-alkanes [33].

The gas products were collected at the end of each stage (Table 1) using a Tedlar bag and analysed off-line using an Agilent 7890A GC and Agilent’s refinery gas analysis method. The instrument had three channels: (i) A HayeSep Q column with a thermal conductivity detector (TCD) to measure H_2_ (N_2_ carrier gas), (ii) a HayeSep Q column with TCD to measure O_2_, N_2_, CO, CO_2_, SH_2_ and C_1_–C_2_ hydrocarbons (He carrier gas) and (iii) a 5A molecular sieve column with a flame ionisation detector to measure C_1_–C_7_ hydrocarbons (He carrier gas).

### 2.5. Hydrotreating Effectiveness and Catalyst Selectivity

The catalyst activity was evaluated in terms of sulfur and nitrogen content of the hydrotreated gas oil. The effectiveness of Ph-HCl-supported catalysts to remove these compounds (i.e., hydrodesulfurisation-HDS and hydrodenitrogenation–HDN) was estimated according to the following equation:

(1) $$HDX\;\left(\%\right)=\frac{\left(X_{0}-\left(X_{p}\cdot ɳ\right)\right)}{X_{0}}\cdot 100$$

where *X*_0_ and *X_p_* correspond to the sulfur (S)/nitrogen (N) content of the feedstock (AGO or AGO/RSO mixtures) and the hydrotreated gas oil (wt.-ppm), and ɳ corresponds to the quotient of the quantity of hydrotreating gas oil produced divided by that of the feedstock processed.

During the co-processing stages, it was also possible to evaluate the catalyst selectivity towards hydrodeoxygenation (*HDO*) or (hydro)decarboxylation/decarbonylation (*DCO*) pathways during triglycerides hydrotreating according to Equations (2) and (3):

(2) $$HDO\;\left(\%\right)=\frac{\sum \left(even\;n–alkanes\right)}{\sum \left(total\;n–alkanes\;\left[even+odd\right]\right)}\cdot 100$$

(3) $$DCO\;\left(\%\right)=\frac{\sum \left(odd\;n–alkanes\right)}{\sum \left(total\;n–alkanes\;\left[even+odd\right]\right)}\cdot 100$$

where ∑(even n–alkanes) and ∑(odd n–alkanes) correspond to the n-alkanes (wt.%) produced via *HDO* (mainly n-C_16_ and n-C_18_) and *DCO* (mainly n-C_15_ and n-C_17_) pathways, respectively, and ∑(total n–alkanes [even+odd]) corresponds to the total n-alkanes (wt.%) formed only by the hydrotreating of RSO triglycerides. These n-alkanes are mainly located in the range n-C_15_ to n-C_18_. This estimation assumes that there is not a significant interaction between the AGO and the RSO during the co-processing stages.

## 3. Results and Discussion

### 3.1. Catalyst Characterisation

As previously described in the Introduction section, the authors have already studied the use of these Ph-HCl catalysts for mesityl oxide reduction [25]. In that paper, these catalytic materials and their main properties were described in detail, including a hydrogen temperature-programmed reduction (H_2_-TPR), ammonia and CO_2_ temperature-programmed desorption (NH_3_/CO_2_-TPD), a scanning electron microscope (SEM) and Energy-dispersive X-ray spectroscopy (EDS) analyses. Thus, the catalyst characterisation is only briefly described in this work, referring to the previous manuscript. Table 2 shows the main results from the catalyst characterisation of Ph raw material, Ph-HCl and Co-Mo/Ph-HCl, Ni-Mo/Ph-HCl, Ni-W/Ph-HCl catalysts.

During the Ph-raw material dealumination with HCl, the Al, Na, Ca and Fe content decreased, which manifested as an increase in S_BET_ (from 7.6 to 120.0 m^2^/g). The authors have well-described this fact in a previous document, together with a detailed analysis of dealumination conditions and phonolite raw material properties changes [21]. In the case of catalysts, the S_BET_ decreased due to the metal deposition during the impregnation catalysts synthesis method (always in a similar way). However, it did not lead to a decrease in the catalyst activity due to active metal sites formation (5 wt.% for promotors–Ni or Co; and 10 wt.% for active metals–Mo or W).

**Figure 3:**
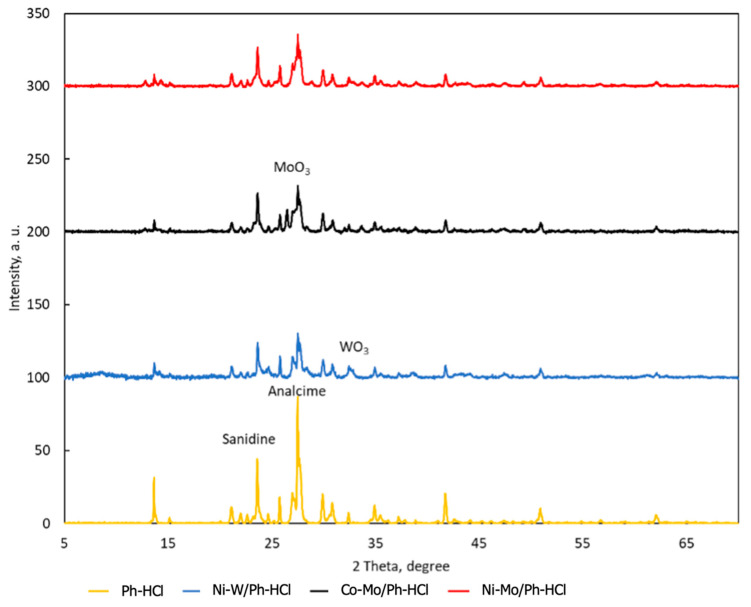
XRD diffraction patterns of Ph-HCl support and catalyst samples (Co-Mo/Ph-HCl, Ni-Mo/Ph-HCl, Ni-W/Ph-HCl) [25].

XRD analysis was used to identify the composition of the main phases of the Ph-HCl catalyst samples. Figure 3 shows the XRD diffraction patterns. This analysis reveals low crystallinity material, with some peaks corresponding to the main phases, i.e., molybdenum and tungsten oxides (indicated in Figure 3). For Ph-HCl, the diffraction pattern was slightly different, identifying the feldspar groups of minerals (sanidine and analcime) [34].

### 3.2. Catalyst Activity for Hydrotreating

The activity of the catalysts was evaluated in terms of the density, sulfur content and nitrogen content of the hydrotreated gas oil obtained during AGO processing and AGO/RSO co-processing. Table 3 shows the characterisation of AGO, RSO and AGO/RSO mixtures used for co-processing (95/5, 90/10 and 75/25). Both feedstocks showed common properties according to their nature (i.e., middle distillate and vegetable oil).

**Figure 4:**
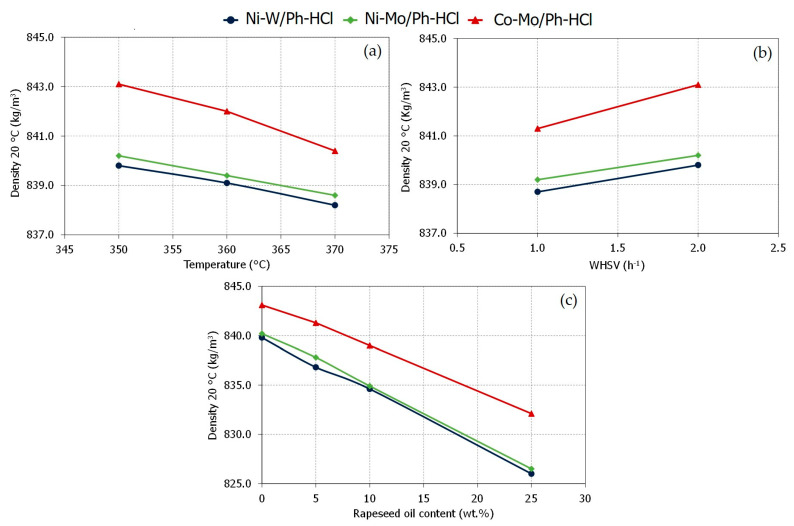
Density of hydrotreated gas oil produced during AGO processing ((**a**): 350–370 °C, WHSV 2 h^−1^, 5.5 MPa; (**b**): 350 °C, WHSV 1-2 h^−1^, 5.5 MPa) and AGO/RSO co-processing ((**c**): 350 °C, WHSV 2 h^−1^, 5.5 MPa, RSO 0, 5, 10 and 25 wt.%). (Note: The 0 wt.% of RSO corresponds to 100% AGO).

During hydrotreating, one of the most common changes in the properties of the hydrotreated product is a change in the density of the organic phase. For sulfidic catalysts, lower densities were related to a higher activity of catalysts [35]. Figure 4 shows the densities of the hydrotreated gas oil during AGO processing at different temperatures and feed rates (350–370 °C, WHSV 1–2 h^−1^, 5.5 MPa), and during AGO/RSO co-processing (350 °C, WHSV 2 h^−1^, 5.5 MPa) after reaching the steady state.

As expected, Ph-HCl-supported catalysts behaved similarly to common industrial hydrotreating catalysts [35]. This behaviour means that an increase in the reaction temperature (Figure 4a) or a decrease in the feed rate (Figure 4b) promotes catalyst activity. This fact manifested as lower values of density for the hydrotreated gas oil obtained during the AGO processing. At the same operating conditions, the Ni-W/Ph-HCl catalyst showed the highest activity (i.e., lowest values of density in the hydrotreated gas oil), which is in response to higher hydrocracking activity of W active-metal with the Ni-promotor in hydrotreating catalysts [36].

**Figure 5:**
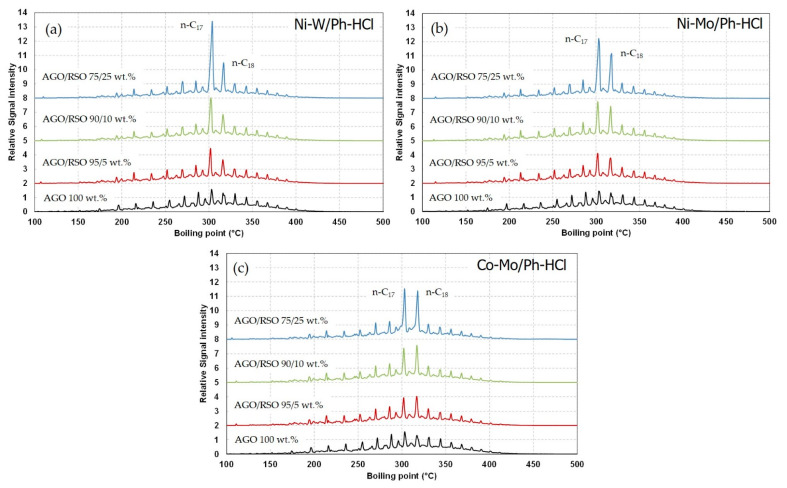
Simdis results of hydrotreated gas oil obtained during AGO processing (350 °C, WHSV 2 h^−1^, 5.5 MPa) and AGO/RSO co-processing (350 °C, WHSV 2 h^−1^, 5.5 MPa) at different ratios (95/5, 90/10 and 75/25) with Ph-HCl-supported catalysts ((**a**): Ni-W/Ph-HCl; (**b**): Ni-Mo/Ph-HCl, (**c**): Co-Mo/Ph-HCl).

During the co-processing of AGO with RSO (Figure 4c), the density of the hydrotreated gas oil linearly decreased with the amount of RSO processed. This fact makes sense due to the complete conversion of RSO triglycerides into n-alkanes (mainly from n-C_15_ to n-C_18_). Those n-alkanes have a lower density than the common components present in the hydrotreated gas oil [37]. Analogous to the AGO processing, the Ni-W/Ph-HCl catalyst again showed higher activity, which may be related to higher hydrocracking activity. As described in the Material and Method section, the conversion of triglycerides was checked by ATR-FTIR and the n-alkanes identified by Simdis. Figure 5 shows the n-alkanes fingerprint during AGO processing and AGO/RSO co-processing.

The formed n-alkanes due to RSO co-processing also indicates the catalyst selectivity of hydrotreated triglycerides (i.e., *HDO* and *DCO* pathways). This selectivity has already been well studied and described in a previous study of the authors, which evaluates the selectivity of Ni-Mo, Co-Mo and Ni-W hydrotreating catalysts during RSO hydrotreating [38]. This set of PH-HCl-supported catalysts has behaved in a very similar manner. Thus, the Ni-catalysts (Ni-W/Ph-HCl and Ni-Mo/Ph-HCl) promoted the *DCO* pathway. This fact means a C-C bond cleavage of carboxylic acids, splitting off the carboxylic group and releasing it in the form of CO_2_, obtaining odd n-alkanes (n-C_15_ and n-C_17_). On the other hand, the Co-catalyst (Co-Mo/Ph-HCl) promoted *HDO* over *DCO*, resulting in higher H_2_O production instead of CO/CO_2_ gases and even the formation of n-alkanes (n-C_16_ and n-C_18_). The absence of peaks at >450 °C also confirms the complete conversion of triglycerides into n-alkanes.

**Figure 6:**
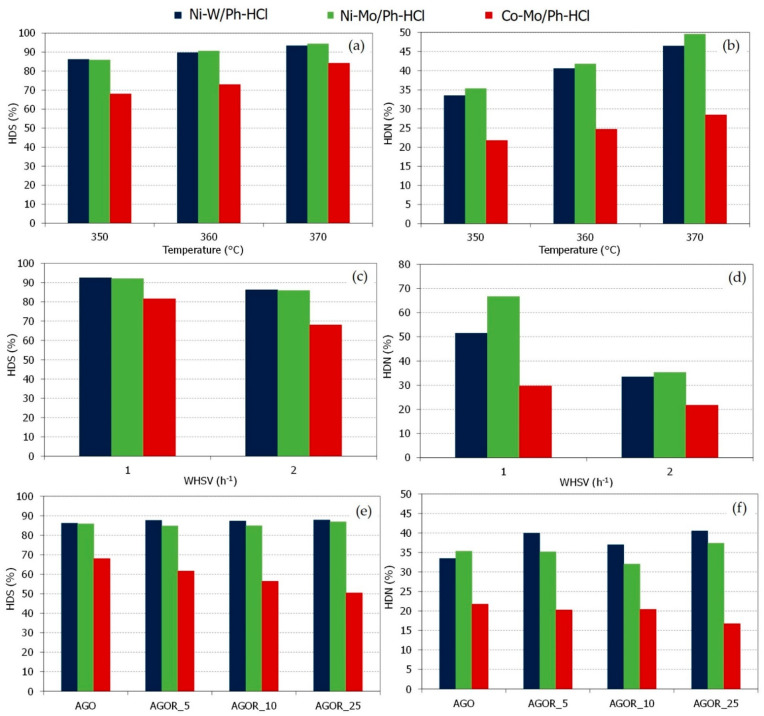
HDS and HDN efficiencies of Ph-HCl-supported catalysts (Ni-W/Ph-HCl, Ni-Mo/Ph-HCl and Co-Mo/Ph-HCl) for AGO processing at different temperatures ((**a**,**b**): 350–370 °C, WHSV 2 h^−1^, 5.5 MPa), WHSV ((**c**,**d**): 350 °C, WHSV 1–2 h^−1^, 5.5 MPa) and co-processing ((**e**,**f**): 95/5, 90/10 and 75/25; 350 °C, WHSV 2 h^−1^, 5.5 MPa).

From the point of view of the sulfur and nitrogen content in the hydrotreated gas oil (i.e., HDS and HDN catalyst efficiencies), the Ph-HCl-supported catalysts also showed similar behaviour to commercial hydrotreated catalysts. Figure 6 shows the HDS and HDN efficiencies, together with sulfur and nitrogen content, during AGO processing and AGO/RSO co-processing.

From the data in Figure 6a–d, it can be seen that Ph-HCl-supported catalysts showed significant catalyst activity comparable with commercial ones [39]. This point means that these catalysts could remove at least 85% and 25% of sulfur and nitrogen compounds present in the AGO, respectively. This ratio was even higher in the case of Ni-promoted catalysts with ratios of HDS and HDN > 90 and >45 %, respectively.

Analogous to the hydrotreating/hydrocracking character analysed by the density of the hydrotreated gas oil (Figure 4), changes in the operating temperature or feed rate affected the catalyst activity. Thus, an increase in the reaction temperature or a decrease in the feed rate meant increased HDS/HDN efficiencies. Nevertheless, it was the impregnation metals used that played the most significant role in catalyst activity. For these hydrotreating and co-processing experiments, the Ni-promoted catalysts showed the highest HDS and HDN efficiencies, especially when MoSx composed the active phase (Ni-Mo/Ph-HCl). These differences in HDS/HDN efficiencies can be explained by considering the characteristics of the catalysts. In this sense, as we described in a previous manuscript [25], the Ni-promoted catalysts (i.e., Ni-W/Ph-HCl and Ni-Mo/Ph-HCl) showed more acid sites, which has been reported as a critical factor related to HDS/HDN efficiencies improving. This fact was similar to the observed for phosphorus-modified metal transition hydrotreated catalysts [40,41].

The effect of co-processing on catalyst activity is shown in Figure 6e,f. Surprisingly, there was no significant decrease in HDS/HDN efficiencies of Ni-promoted catalysts, maintaining the same range of activity previous to co-processing (86–87% and 35–40% for HDS and HDN efficiencies, respectively). This result points to better stability of Ph-HCl supported catalysts regarding common Al_2_O_3_/SiO_2_-supported commercial catalysts [42], which usually shows a higher activity decrease when co-processing. However, this was not the case with the Co-Mo/Ph-HCl catalysts, with catalyst activity that decreased with the amount of the RSO co-processed (from 68 to 50% and 22 to 17% for HDS and HDN efficiencies, respectively). The catalyst deactivation during co-processing could be due to several factors such as sulfur leaching from the catalytic surface or coking [16,43]. Moreover, Co-Mo catalysts have been reported as more sensitive to oxygen compounds due to active site inhibition by the effect of CO/CO_2_ [44]. That is the reason Ni-promoted catalysts are preferred in cases of co-processing [45].

## 4. Conclusions

Three acid-treated phonolite-supported Ni-W, Ni-Mo and Co-Mo catalysts were synthesised and tested to hydrotreat atmospheric gas oil and co-processing of rapeseed oil at standard industrial conditions (350–370 °C, WHSV 1–2 h^−1^ and 5.5 MPa). The catalytic behaviour of these catalyst samples was similar to commercial hydrotreated catalysts supported by Al_2_O_3_ or SiO_2_. This point means significant hydrodesulfurisation and hydrodenitrogenation efficiencies (up to 90 and 50 %, respectively) during the processing of atmospheric gas oil, as well as a complete conversion of triglycerides during the co-processing of 25% of rapeseed oil. For Ni-promoted catalysts (i.e., Ni-W/Ph-HCl and Ni-Mo/Ph-HCl), the significant activity was accompanied with strong stability during co-processing, without an apparent activity decrease due to oxygen-compounds addition, common in conventional catalysts for hydrotreating. Our results enabled us to evaluate the potential of phonolite as supporting material for hydrotreating catalysts, pointing to the best catalyst performance for middle distillates and vegetable oils. This work is our next step in developing promising catalysts to produce the necessary platform chemicals or mandatory biofuels, which is in line with the current energy challenge targets and EU demands.

## Figures and Tables

**Table 1:** Description of reaction conditions in chronological order.

Stage	Feed	TOS (Time on Stream, h)	Temperature (°C)	WHSV (h^−1^)	Pressure (MPa)
1	AGO	0–60	350	2.0	5.5
2	AGO	60–84	360	2.0	5.5
3	AGO	84–108	370	2.0	5.5
4	AGO	108–132	350	2.0	5.5
5	AGOR_5 ^1^	132–156	350	2.0	5.5
6	AGOR_10	156–180	350	2.0	5.5
7	AGOR_25	180–204	350	2.0	5.5
8	AGO	204–228	350	2.0	5.5
9	AGO	228–276	350	1.0	5.5

^1^ AGOR_X means a mixture of AGO with RSO where X = wt.% of RSO.

**Table 2:** Characterization of Ph raw material, Ph-HCl, Co-Mo/Ph-HCl, Ni-MoPh-HCl and Ni-W/Ph-HCl.

Sample	Ph (Raw Material)	Ph-HCl	Ni-W/Ph-HCl	Ni-Mo/Ph-HCl	Co-Mo/Ph-HCl
Specific surface BET, m^2^/g	7.6	120.1	68.3	41.8	51.3
Pore volume (3–50 nm), cm^3^/g	0.003	0.030	0.016	0.000	0.000
Total intrusion volume, cm^3^/g	0.008	0.179	0.118	0.200	0.209
XRF Elemental Analysis, wt.%	-				
Si	26.5	34.8	28.5	28.9	29.6
Al	11.8	6.7	5.3	5.5	5.7
Ni	0.0	0.0	5.2	5.4	0.0
W	0.0	0.0	9.8	0.0	0.0
Mo	0.0	0.0	0.0	10.0	9.0
Co	0.0	0.0	0.0	0.0	4.6
Na	7.9	2.8	1.7	1.2	1.4
K	5.1	6.6	5.2	2.8	2.7
Fe	1.4	0.8	0.5	0.6	0.6
Ca	0.7	0.0	0.0	0.1	0.1

**Table 3:** Characterisation of used feedstocks for processing and co-processing.

Property	AGO	RSO	AGO/RSO 95/5	AGO/RSO 90/10	AGO/RSO 75/25
Density at 20 °C (kg m^−3^)	852.6	914.5	856.0	859.0	868.1
Refractive index at 20 °C	1.4759	1.4756	1.4759	1.4759	1.4758
S content (ppm)	11,010.0	2.3	10,500.6	9910.2	8560.1
N content (ppm)	239.0	22.9	228.2	217.4	185.0
Acid number (mg KOH g^−1^)	0.04	0.10	-	-	-
Elemental analysis (%)	-				
Carbon content	86.0	78.6	85.6	85.3	84.2
Hydrogen content	13.3	12.0	13.2	13.2	13.0
Simdis (wt.%)	-				
10	220	595	-	-	-
30	281	608	-	-	-
50	308	609	-	-	-
70	335	610	-	-	-
90	373	612	-	-	-

## Data Availability

Data sharing not applicable.
